# Remarkable Operation

**Published:** 1881-12

**Authors:** 


### REMARKABLE OPERATIONS.

Among the remarkable operations performed in Germany recently by eminent surgeons, those in which the stomach or abdomen had to be opened, have been at once the most dangerous and the most successful. After the achievements of Dr. Billroth, of Vienna, in the removal of cancers from the stomach, comes now Dr. Schinzenger, a Professor in the University of Freiburg, with two cases in which obstruction in the entrails had to be removed. In one case, that of a woman, a section of one of the intestines had to be cut out and the severed end sewed together. In both instances speedy recovery followed.

